# How do nurses and midwives perceive their role in sexual healthcare?

**DOI:** 10.1186/s12905-022-01891-y

**Published:** 2022-08-04

**Authors:** Mathilde Azar, Thilo Kroll, Caroline Bradbury-Jones

**Affiliations:** 1 Faculty of Health Sciences, University of Balamand, Beirut, Lebanon; 2 School of Nursing, Midwifery and Health Systems, University College Dublin, Belfield, Dublin 4, Ireland; 3 School of Nursing, University of Birmingham, Edgbaston, Birmingham, B15 2TT UK

**Keywords:** Sexual health, Nurses, Midwifery, Qualitative research, Focus groups

## Abstract

**Background:**

Nurses and midwives role in sexual healthcare is essential to help patients, particularly women, ensure a satisfactory sexual wellbeing. Yet, these professionals often overlook this aspect of patients’ health. Little is known regarding nurses and midwives’ attitudes, views and experiences concerning sexual healthcare. Using a naturalistic inquiry approach, this qualitative study was conducted to overcome this limitation and gain insights into nurses and midwives' role in the delivery of sexual healthcare.

**Methods:**

A purposive sample of nurses and midwives was chosen from different clinical sites. Data generated by focus group discussions were were analysed using the Framework Analysis while adopting different strategies to ensure rigour. The study aligns with the consolidated criteria for reporting qualitative research checklist.

**Results:**

Five themes illustrated the participants’ views and experiences. These are: ‘Perceptions of sexuality’, ‘Appreciating the discussion around the individuals' sexual issues’, ‘Muting the discussion around the individuals’ sexual issues, ‘Coping with embarrassment’, and ‘Promoting nurses’ and midwives’ roles sexual healthcare’. Nurses and midwives discussed the importance of sexuality in the couple's life. They reported controversial views and highlighted many challenges that make them reluctant in playing an efficient role in sexual healthcare. They discussed many suggestions, mainly getting a solid sexual health education to become better equipped to meet patients’ sexual health needs.

**Conclusion:**

Findings are critical to empower nurses and midwives, break the barriers in discussing sexual healthcare and integrate this aspects of care more actively and confidently in daily practice.

## Background

Sexual health is an indicator of peoples’ physical, emotional, mental and social wellness. It is not only about the practice of sex; it is an integral part of the personality, identity and self-affirmation [1]. Sexuality is a meaningful and enriching personal experience. It prevents many adverse health outcomes and enhances people’s well-being [2]. Sexuality is a right to respect and protect and a need to attend to and fulfil without any discrimination or judgement [3].

Many patients claim the importance of their sexual health and have questions and concerns that are not addressed. There is a widely held belief that for people who are ill or older, sexual functioning and sexuality are not a priority [4]. Consequently, their sexual needs often remain unfulfilled.

As part of the holistic and patient-centred care, nurses and midwives play a crucial role in assessing the individuals’ sexual health and providing counselling, clinical services and referral to specialists as needed [5]. However, the contributions of these professions have yet to be fully recognised and realised [6, 7].

Sexual health is often adversely affected by physical and psychosocial problems which may affect sexual functioning and lead to sexual dissatisfaction. For example, more than half of diabetic men and women suffer from sexual dysfunction [8]. This problem is serious among patients with chronic kidney disease, hemodialysis [9] and cardiovascular problems [10]. Breast and prostate cancer, the most prevalent females and males’ cancer, heavily alters patients’ sexual life [11, 12]. More than half of the patients with rheumatoid arthritis report different types of sexual dysfunctions [13]. This proportion varies between 33 and 42% respectively among women and men who have mental health problems [14]. Other life conditions like infertility problems among couples, ageing, hormonal changes, chronic diseases and medications are additional risk factors for sexual dysfunction [15, 16]. In turn, sexual dysfunction leads to poor physical and mental health outcomes [17, 18]. It also negatively impacts the quality of life of the partners and impairs family interaction [19].

For these reasons, it is important to give peoples the opportunity to express their sexual concerns and receive the necessary assistance. According attention to sexuality in the plan of care is a key element of sexual health promotion and improvement. Therefore, the availability, accessibility and affordability of sexual healthcare become a must.

Indeed sexual health is becoming more prominent; yet, it is still overlooked in light of other health priorities and the existing services are not used to the best of their advantages. Many nurses believe that sexual health is not as important as other medical problems [17, 20]. They even ignore the importance of tackling the topic with the patients [5]. In the main, sexual health tends to focus on reproduction and prevention of sexually transmitted infections. The effect of diseases and health conditions on patients' sexual functioning and wellbeing is not considered. Nurses and midwives, seldom discuss this topic with patients, and sexual health is not part of the culture of the clinical practice [17]. They might feel ill-equipped and lack the preparedness and qualifications to address such a taboo and private topic [4, 17]. Nurses assume that patients do not expect them to initiate sexual discussion or that sexual assessment is not a priority for an ill patient [21, 22]. Yet, the patients feel more comfortable if the professionals encourage them to express their sexual concerns [23–25].

A paradox exists between the high prevalence of sexual problems and nurses and midwives’ reluctance to provide sexual healthcare. The WHO [1] confirms that sexual health information and promotion are integral parts of primary healthcare services. Nurses and midwives should develop their competence and overcome the barriers that prevent them from having an active and effective sexual healthcare role [17, 26].

In Lebanon, studies about nurses’ and midwives’ role in sexual health do not exist. Sexual health assessment and management seldom feature in a plan of care. In parallel, people cannot easily talk about their sexual issues and discuss them with the healthcare providers [27]. Consequently, people are not assisted and do not have access to specific and adequate answers to their sexual concerns [28]. This would probably affect their sexual health and overall wellbeing as sexuality is an important indicator of the quality of life which is more commonly addressed nowadays [29].

The majority of the studies about the topic are epidemiological and have a narrow focus on the barriers that prevent sexual healthcare. Qualitative research that report on nurses and midwives views are scarce. Understanding nurses and midwives perception and experience of sexual healthcare and highlighting the challenges related to their role are crucial to promote sexual health. As findings are grounded in the Lebanese healthcare system and culture, they will be helpful to realistically address the problem and support nurses and midwives in offering sexual health services.

## Methods

This study aims at exploring the experience of nurses and midwives in assessing patients’ sexual health issues and concerns and the perceived factors that affect their role in the delivery of sexual healthcare. The focus of the participants’ discussion was on female patients as all of them are females and feel more comfortable addressing sexual issues of patients with the same social gender.

An exploratory, descriptive study was used. This qualitative design generated data that uncovered the professionals' attitudes, perceptions and experiences. It was suitable to get insights into this unexplored and sensitive subject and the reported factors behind sexual healthcare practice. This study aligns with COREQ, the consolidated criteria for reporting qualitative research [30].

### Sampling and participants

The sample included 11 participants, four midwives and seven nurses, selected from one university hospital and two primary healthcare centres in Lebanon forming a sample of clinical and community professionals. The sample was purposefully chosen to reflect the viewpoints of nurses and midwivesfrom different backgrounds, working in diverse clinical areas with people experiencing a range of health conditions. This sampling approach generated rich data that enhanced the credibility of findings. An invitation letter was sent to the professionals through the nurse manager informing them about the study and the ethical considerations. A sample of participants who accepted to get involved in the study was purposively selected considering their education, area of work, years of experience and marital status, ensuring variation and rich data generation. All registered nurses and midwives who had direct patient care were eligible to participate. The characteristics of the sample are presented in Table 1 below.

**Table 1:** Sociodemographic characteristics of the participants

Pseudonyms	Education	Area of work	Years of experience	Marital status
Noel	Midwifery Diploma	Maternity	3	Single
Melissa	Midwifery Diploma	Maternity	5	Single
Adrea	Nurse [BSN]	Medical/surgical	18	Married
Sabrine	Nurse [MA psychology]	Oncology	3	Single
Zovinar	Nurse [BSN]	Outpatient clinic/OPC	3	Single
Damy	Nurse [BSN]	Psychiatry	15	Married
Angy	Nurse [MSN]	Oncology	6	Married
Rayan	Midwifery Diploma	Maternity	5	Single
Nahla	Clinical nurse specialist	Palliative care	20	Married
Jennifer	Midwifery Diploma	Maternity and OPC	10	Single
Rea	Clinical nurse specialist	In-service education	12	Single

### Recruitment and data collection

The primary investigator, MA, conducted all the fieldwork. She recruited the participants and conducted two focus groups of six and five participants respectively. This took place between 2016 and 2017. In addition to her skills and self-confidence in qualitative interviewing, she is a midwife and has many years of clinical experience. She is familiar with the topic and shares with the participants the same language. Moreover, she is aware of the specificity of the Lebanese healthcare system and the limitations regarding sexual health practice. During the recruitment process, MA provided nurses and midwives participants with information about the study objectives, their voluntary participation and the confidentiality of data. She gave them time to thoroughly read the consent form and answer their queries before signing it. This first encounter was an occasion to establish a trustful and comfortable relationship with the participants and get easily engaged in the group discussions.

With the agreement of the participants, MA conducted two focus group discussions composed of five and six participants each, in a private and comfortable setting at the University where she works as a full time faculty. She ensured a friendly environment to encourage the participants to freely express their ideas without fear of any judgement. She stressed on the importance of sharing and discussing their views spontaneously to get insights into their perceptions and experiences and understand the factors that may affect their role in sexual healthcare. A note-taker, nurse instructor, assisted the primary investigator. She provided logistic support, collected data about the participants’ impressions, attitudes, interactions and verbal and nonverbal body language, commented on the general environment of every focus group and reflected on her own thoughts and impressions.

In addition to the sociodemographic data, a topic guide was developed and included the following semi-structured interview questions and prompts:

1. *What has motivated you to take part in this study?*
2. *What do you understand by having a good sexual life?*
3. *What is your role with the individuals who express sexual concerns or problems?*
  *Prompt: How do you assist them? What are the initiatives you take?*
4. *Could you describe any 'practice as a nurse or midwife' where you had to deal with an individual who expressed sexual concerns or problems?*
  *Prompt: What did you do? How did you address/manage the situation? To whom did you refer for help? Was this person satisfied? Explain*
5. *What factors may interfere with the assessment and discussion of the individuals' sexual health issues?*
  *Prompts: Personal, organisational, cultural*
6. *What would you suggest could be changed to improve your role in sexual healthcare?*

These mostly open-ended questions allowed the participants to extensively discuss the different aspects of their role and the problems encountered in delivering sexual healthcare at the personal, professional and organisational levels. The questions were tested with one midwife and one nurse to ensure the clarity and pertinence. Minor adjustments were introduced.

The focus group discussions took 2–3 h and were audio-recorded after the approval of the participants. The discussion was very dynamic and interactive, showing participants enthusiasm in expressing and arguing their ideas and differing views. They extensively reflected on the different dimensions of the subject and provided very rich information embedded in their varied backgrounds. At the end of every focus group, the main ideas were summed up, and the participants were asked if they had anything to elaborate, change or omit. Nobody had any additional input. Immediately after the interviews, MA documented the field notes to avoid missing any details. She included all verbal and non-verbal cues relevant to data analysis and interpretation, her impressions, thoughts and preliminary interpretations of the interviews. She then compiled her notes with those collected by the note-taker after a common discussion and debriefing.

Data saturation was determined after the two focus groups as the participants were highly engaged and talkative. They provided thick (quantity) and rich (quality) descriptions of the topic. There was no need to have more coding to replicate the study and ensure the transferability and credibility of findings.

### Data analysis

Each interview was transcribed verbatim as soon after the interview as possible, and the field notes were added to the margin of the transcripts. Data analysis was informed by Framework Analysis: A qualitative methodology for applied policy research [31]. This involved a five-stage process that is structured, moving back and forth from raw descriptive data to a higher level of abstraction and construct a meaningful conceptual frame. The three researchers were involved in the data analysis.

The first stage was characterised by critical listening to the tapes and thorough reading through the transcripts to get immersed in the data context. Accordingly, the codes were identified as they derived from the data. Next, a thematic frame was created and served to classify data under different themes and subthemes loosely. In the indexing stage, the coded themes and subthemes were introduced alongside textual data as appropriate. Then, all data across the different transcripts were cut and pasted to a chart according to the created themes and subthemes. All raw data were translated from Arabic, the native language of the participants, to English using the back-translation method. This allowed the three researchers to verify the codes, organise the raw data, classify them under different themes and subthemes, and compare and contrast findings. The researchers further sifted, summarised, and synthesised data supported by relevant quotes that illustrate the participants' views at the last stage. A thematic framework of the data is presented in Table 2 below and an example of the analysis process.

**Table 2:** Thematic framework of the data generated with the participants

Theme	Sub-themes	Codes
*Perceptions of sexuality*	Value of sexual life	*When it is healthy/good, everything is healthy/good*
	Male sexual leadership	*Women in the Arab world do not orgasm*
	Poor sexual education	*We receive couples who know nothing about the genital anatomy and how to have penetrative sex* *… the problem relates to the absence of proper sexual education*
*Initiating the discussion about the individuals’ sexual issues*	Establishing a suitable relation	*… you initiated the conversation… you allowed the patient to talk…*
	Trigger the patients to talk	*Every day during my morning rounds, I interview every patient and ask him/her about his/her concerns. Otherwise, the patient does not talk…*
*Muting the discussion around the individuals’ sexual issues*	Sexual healthcare is not part of the culture	*We learned in our upbringing that the topic is intimate. That is why we have not yet reached the point where we can easily approach it or consider it a concern like other topics*
	Lack of knowledge	*We were not informed in our childhood and when we got older and we did not learn… I can’t do it…*
	Taboo	*I answer if I am asked. But to do it by myself! No*
	Not a priority for ill patients	*Of course, yes; not easy on her; losing the breast! Of course it is even traumatising. But still, there are issues more vital. Does she care [about sex] while her life is threatened?*
*Coping with embarrassment*	Delegating sexual healthcare	*I am sure that he is better [the physician]… these issues, too complicated… He knows, he knows things well, he is familiar with the couples…*
	Avoiding sexual healthcare	*For my part, I am shy. If I ask her, she will notice my perplexity. For us also, the subject is taboo*
*Promoting nurses' and midwives' role in sexual healthcare*	Suggestions to deal with the barriers	*Personal, educational, professional, and social*

### Rigour

Using trustworthiness criteria of Lincoln and Guba for qualitative research, we put in place the following steps to ensure rigour [32]. Credibility was achieved using audio-recording to accurately and integrally secure data. In addition, a log trail served as a researcher reflexive practice to minimise bias and a way to track all the decisions and show transparency. Transferability was achieved through clear identification and variation of the sample, setting, and thick data description. Dependability was ensured by referring to the field notes and tracking all the decisions made throughout the research process. The three researchers separately analysed the two transcripts and then compared findings until a consensus was reached. Strategies used to enhance the confirmability of the study were auditing, debriefing and discussion of findings with external peers. The raw data were in Arabic. A translation back-translation was meticulously conducted to maintain as authentically and accurately as possible participants' ideas.

### Ethical consideration

All methods were carried out in accordance with ethical guidelines and regulations**.** Before data collection, we obtained the Institutional Review Board from the University of Balamand, Faculty of Medicine and Medical Sciences, and Saint George Hospital University Medical Centre. The access to the participants required the approval of the concerned healthcare centres. All the participants made an informed decision about their involvement in the study and a signed informed consent was obtained from all of them ascertaining their voluntary participation and the confidentiality of findings that will be only accessible to the researchers and used for scientific purposes.

## Findings

The purpose of the focus group discussions was to answer the two research questions:

- What is nurses and midwives experience in sexual healthcare and how do they perceive their role?
- How is nurses and midwives' role in sexual healthcare affected by the perceived personal and sociocultural beliefs as well as the healthcare system?

**Fig. 1:**
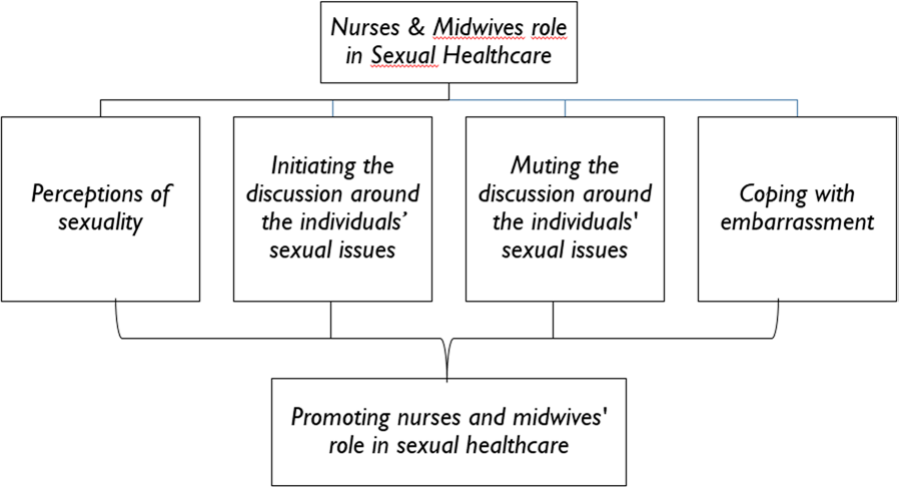
Findings about nurses’ and midwives’ feedback on their sexual healthcare role

As illustrated in Fig. 1, data reported by nurses and midwives are presented under five themes: ‘Perceptions of sexuality, ‘Initiating the discussion around the individuals’ sexual issues’, ‘Muting the discussion around the individuals’ sexual issues, ‘Coping with embarrassment’, and ‘Promoting nurses’ and midwives’ role in sexual healthcare’.

### Perceptions of sexuality

At the opening of the discussion, participants were asked about their views concerning sexuality, believing that the way they look at the subject affects their practice. Almost all of them seemed to be challenged by the question and did not easily verbalise their thoughts. The majority of the midwives and married nurses looked more confident than their other colleagues in initiating the discussion.

Participants valued sexual life for the individuals' and couples' wellbeing. They described it as a 'boost of energy and 'mental therapy' that stimulates the person and gives him/her a sense of welfare about the self and the partner. Their common stated that a '*good sexual life is good for a positive life overall'* within a relationship based on communication and transparency. For example:

*As a married woman, I will start (reflecting on the participants' hesitance to start the talk). I feel that sexual relation, a healthy/good relationship between a man and woman, starts with the sexual interaction and continues throughout other issues. (Nahla, Clinical Nurse Specialist/CNS, palliative care).*

While participants were vocal in advocating for reciprocity and equality in a couples' relationship, they associated a sexual leadership role to men paralleled with women's poor sexual experience as a result of gender discrimination. They assumed that most women do not orgasm, and their difficulties are relational and psychosocial rather than physical.

The absence of men and women's proper sexual education was identified by all the participants as another sexual concern. Based on their clinical practice, the midwives described the cases of many couples who struggle with a variety of sexual problems because of their misconceptions, lack of knowledge and inhibition. Zovinar, a community nurse, highlighted the need for sexual awareness by saying:

*If you are prepared for sexual changes and every phase (little girl, teenager, adulthood, menopause), you would have a more appropriate response (Zovinar, BSN nurse, Outpatient Clinic/OPC).*

Participants agreed that sexual concerns are highly prevalent but are underreported. Many of them identified the need to break the taboo around sexuality; others seemed reluctant to engage in the field. How the role of these professionals is conceptualised and affected is presented below.

### Initiating the discussion around the individuals’ sexual issues

In general, the professionals acknowledged the importance of discussing the individuals’ sexual concerns as many diseases and therapies affect sexual function and make people worried and in need of information and support. Therefore, a relationship based on attentive listening, time commitment and dedication in a private, trustful and non-judgmental environment was deemed warranted to respond to their sexual needs.

*Because someone came, broke the ice with the patient and triggered the patient to talk… (Rea, CNS, In-service education).*

*Initially, this discussion could not be broached unless you trust the person in front of you and you are sure that this person will listen to you and keep your discussion confidential (Nahla, CNS, palliative care)*

The majority of participants, especially those who were engaged in community health (Nahla, Zovinar and Jennifer), had long years of experience (Adrea) and were assigned staff development responsibilities (Rea), defended their position as caregivers, educators, and advocates for people. They expressed their willingness to have a fundamental role in sexual healthcare. Rea suggested incorporating sexual assessment in the physical exam as she is a specialist in the domain.

The majority agreed and valued sexual assessment as a first step to appropriately address women's sexual concerns through interdisciplinary teamwork.

*We will not solve all the problems of women, I am sure. But at least, we, in our way as women, can let women talk to help them and address their needs as appropriate. We have a referral role, and we have to work as a team to solve women's problems (Jennifer, Midwife, inpatient Clinic/IPC & Outpatient Clinic/OPC).*

Others had opposing views and negative attitudes, thus further muting individuals’ sexuality.

### Muting the discussion around the individuals’ sexual issues

The participants reported that sexual health is not part of nursing and midwifery culture, criticising the curricula that merely address the subject.

*It is a taboo topic. It is seriously taboo for us also. We, how we were brought up? We were inhibited before we entered universities and learned. We took 15 hours of sexology; nurses take less. Are 15 hours enough? Not at all. (Jennifer, midwife, IPC & OPC).*

Melissa, a midwife, was against the idea of broaching the discussion about sexual issues with the couples as this was intimidating and useless.

*To open the discussion about sexuality!? No, I do not feel comfortable doing it. Initially, I do not think this kind of talk is important within the context of the hospital (Melissa, midwife, maternity).*

Some participants explained that sexual healthcare is a stigmatising; thus, it is up to the patients to broach the discussion if they have a problem; otherwise, it is an intrusion in their private life.

All participants concurred that since sexual healthcare is not integrated in the healthcare system, sexual health assessment and management will remain an individualised initiative that depends on the qualifications and priorities of every health professional.

The oncology and psychiatric nurses were the most reluctant even though their sexuality might be altered. This is exemplified by Damy’s account:

*I do not feel that my role in sexual health is as important as my role in the medication, observation, high blood pressure … I do not care if the patient's sexual needs are fulfilled when there are other more pressing issues… Frankly, I do not care even if I might be a bit rude… (Damy, psychiatry nurse).*

Damy expressed her opinion with determination presuming that the sexuality of psychiatric patients is not addressed on the floor. However, Adrea, a nurse on the medical-surgical floor, had an opposite view. She contested those who were against sexual healthcare by telling the story of a dying woman who expressed her sexual worries. She narrated:

*Woman: Do you think that he (her husband) still looks at me like before?*

*Adrea: Sure he does. He does not leave you alone, even for a minute. What do you feel?*

*Woman: I do not know. I feel that man's interest is in his belly and what is just below his belly.*

*Adrea: Maybe, but what do you feel?*

*Adrea said that she started crying.*

*Woman: Do you think that he goes out?*

*Adrea, in addressing her talk to the group members: Did you see what her worries are? But she was dying. She was crying because she was unable to respond to her husband's needs.*

The participants who work in the hospital identified many challenges inherent to the healthcare practice. They stressed the influence of (a) the workload that does not allow them to give priority to sexual health in regard to other pressing issues; (b) the short stay in the hospital that is not suitable to forge a trustful relation for sexual discussion; (c) the healthcare system where every patient refers to his/her physician to address such a sensitive subject; and (d) the lack of discretion in the hospital. Participants also reflected on their lack of power within a private healthcare system dominated by physicians. This was assumed to affect their social image that is not well recognised and adequately valued, whereas the physician is more knowledgeable, competent and trustworthy.

In comparison to their practice in the hospital, the professionals identified an expanding nursing and midwifery role in the community where they have more potential and authority to act in a systematic, holistic and contextual approach with the patients and specifically with women.

*This is the idea; in the community, the nurse has the right to do these things. These are part of her work… She can work systematically with every family (Sabrine, oncology nurse, MA psychology).*

The use of different coping strategies counteracted nurses' and midwives' embarrassment of sexuality-related care.

### Coping with embarrassment

To escape their embarrassment, many nurses delegated sexuality-related care to the midwives believing that it is the core of their profession or else to the physician. However, the midwives often rely on the gynaecologist to take charge of this responsibility as their work mainly focuses on reproductive health priorities. Unexpectedly, three out of eleven participants asserted that they could not identify with sexual healthcare and refused to recognise this role as part of their scope of practice. For instance, Sabrine's seemingly rigid position stimulated a debate among the members of the group.

*Sabrine: I am against the nurse who takes this role.*

*Noel: I do not agree with you.*

*Rea: Your role is not limited to only support; it is broader than that.*

*Sabrine: You do not have the right to give your opinion.*

*Melissa: You have to have years of experience; not someone newly graduated.*

*Sabrine: How many of us are prepared for sexual healthcare?*

*Rea: It is a justification for the lack of knowledge. Do training, and in this way, you will know…*

*Noel: Why the physician should have a role and we should not?*

*Sabrine: But the nurse does not have the right to give her opinion concerning this topic.*

Avoidance was another tactic adopted by the participants to escape their timidity and social stigma, as Noel recounted:

*You know that even as a midwife, you cannot sometimes talk; they think that you are sexually experienced, and this is socially unacceptable as a single woman (Noel, midwife, maternity).*

Humour was identified as another way to cope with intimidation. For instance, Damy described her embarrassment when a man complained to her about the sexual hyperactivity of his bipolar wife. To escape this bleak situation, she made a joke about the husband's talk and left the room.

*One day I was taking the family history with the husband of a woman who had severe mania. What is the problem? He answered, 'She does not leave me' (her excessive need for sex). Me!!! (She was so surprised) Mr! Sorry, Mr.*

*Every second, every second (continued the husband). Here, for sure, I became red… I told him, 'you are abused?' I did it just to reduce my embarrassment… then. I changed the conversation and avoided more details about the subject with him because I was so shy. I made fun of it and left the interview room. However, during that time, you cannot imagine how much he was embarrassed, and I was intimidated! (Damy, psychiatry nurse).*

To justify her behaviour, Damy reflected on her timidity with her husband, where she never approaches him if he does not take the initiative, asserting that she is copying her parents' life and cannot change.

### Promoting nurses' and midwives' role in sexual healthcare

Talking about the general context of nursing and midwifery practice, the majority of the participants acknowledged the neglect of this aspect of their practice. Conscious of this professional deficiency, they discussed many strategies to promote their role. Their main suggestions were:

- Overcome the timidity and taboo around sexuality by providing sexual education at an early age.
- Have a solid professional preparation to attend to the emerging sexual healthcare needs.
- Encourage the specialisation in the field of sexology to deliver more efficient care.
- Integrate sexual healthcare in the daily clinical practice focusing on sexual health assessment and counselling within interdisciplinary teamwork.
- Develop sexual health centres that are qualified, easily accessible and affordable to encourage counselling and helpseeking.
- Raise public awareness about sexuality and sexual health concerns.
- Empower people to raise their sexual concerns.

Examples of nurses' and midwives' narratives are:

*Nahla. Sexual education should start at school like what Jennifer said. The mother, daughter and son; I mean since the early age…*

*Jennifer. They can do awareness campaigns for the parents to prepare them.*

*Rea. Very important to target women… support them… inform them…*

*Angy. I think that the specialty is very important. A multidisciplinary team is indeed very important, but if not prepared? I prefer to have one competent person who can deal with these things with the patient rather than having 100 persons with different opinions… there should be someone knowledgeable who likes to discuss these things… If you do not like the field, you cannot do it…*

## Discussion

The findings of this study suggest that nurses and midwives situate sexuality in a multidimensional context and admit its importance for individuals' wellbeing. Nevertheless, they have differing views concerning their role in sexual healthcare. A range of reasons were discussed for this discrepancy matching their personal, professional and contextual backgrounds.

Participants framed sexuality within a heterosexual relationship governed by love, understanding, exchange, transparency and respect. They perceive sexuality as an indicator of the individuals' fulfilment, security and happiness even if this is not translated into their daily practice. It might be that nurses may consider the broad aspect of sexuality to avoid discussing its erotic character. This is plausible given that the apparent perplexity and embarrassment of many participants in verbalising their opinions and the hot debates and contradictory thoughts they had during the group discussions around sexual health assessment.

All nurses and midwives recognise the impact of the disease and therapy on sexual function. Nevertheless, those involved in educational responsibilities and community care show more interest and proactiveness in integrating sexual healthcare in daily practice and are more self-confident in discussing the topic. Nurses and midwives are frequently the health professionals to whom patients can refer to get advice and support as they are in the ideal position in the healthcare team to deal with sensitive issues such as sexuality [18].

As reflected by the participants, the taboo and stereotype around sexuality, the lack of knowledge and communication skills, and the conservative attitude persist and limit their role in sexual healthcare.

Similar findings were identified by previous studies making the approach intimidating and stressful for the patients too [6, 7, 33, 34].

Adding to that, interviewed participants are females and face significant cultural limitations to discuss sexual issues with males. This has also affected Jordanian nurses' attitudes and beliefs towards patients' sexual health [35]. Conversely, the study of Klaeson and colleagues revealed that female nurses were more at ease discussing sexual issues with male rather than female patients [36]. Bearing in mind the importance of sexuality for the individuals’ wellbeing may push nurses and midwives to be more considerate in sexual healthcare.

Nurses and midwives' misperception of their role is paralleled with the belief that critically ill patients are not sexually active, particularly when they have oncology and mental health problems. Similar views were reported by other studies [5, 20, 37]. While the condition and treatment can impair libido and energy levels, it does not uniformly impact the patient's ability to enjoy sex or to be interested by sexual life which could be expressed in different ways that go beyond the pure physical satisfaction and sexual intercourse.

On the contrary, having sex and intimacy can have beneficial effects on patients' and partners' relations. Patients with chronic health conditions may have serious sexual concerns and express the need for assistance which is often not provided [38]. Health professionals caring for cancerous and end-stage kidney disease patients ascertain that all those who receive treatment that impacts their sexuality should receive sexual rehabilitation and assistance [18].

In support of other findings [17], many participants avoided invading patients' sexual life. The literature states that the patients feel more comfortable if professionals encourage them to express their sexual concerns; yet, professionals are inclined to wait for the patients to do so [17, 25, 35]. It has been suggested that professionals and patients should be prepared to discuss the topic [38], thus creating a more open environment.

Participants timidity and inhibition do not allow them to be comfortable in sexuality care; they rather adopt a conservative attitude. Being shy and embarrassed and lacking the vocabulary to express their thoughts, it is suggested that some Iranian women and health providers use euphemisms like 'thing' to reflect on 'sex' and 'sexual intercourse' [39].

Not being comfortable with their sexual selves, it becomes difficult to address their patient's sexual issues [18, 40]. On the other hand, studies indicate that nurses who are aware about their sexual-self are more likely to discuss with the patients their sexual concerns and deliver a good quality of sexual healthcare [41, 42].

In addition to the personal limitations the participants face in dealing with the patients sexual concerns, the cultural norms play a major influence. Sexuality is understood as part of the social life; it is governed by the implied sociocultural rules and politics that determine humans’ attitudes and behaviours. Nurses and midwives are affected by these rules and do not seem to break the conformity to the social values and norms even if they are conscious of the importance of sexual healthcare.

The participants criticised their curricula that reflected a conservative stance and did not prepare them to provide adequate sexual healthcare. Similar complaints have been reported in the literature, asserting the need to reinforce a practice-based education that strengthens knowledge and skills [18, 43].

Organisational factors emerge as additional barriers to provide proper sexual healthcare offered in a favourable work environment that is characterised by privacy, good patient-nurse relationships and communication as the topic is complex and time-consuming [25, 41].

Missing the personal and contextual support to provide sexual healthcare, nurses and midwives use different coping strategies to escape their embarrassment. For example, they might delegate this role to other midwives or physicians, avoid discussing the topic, or make a joke of patients' sexual concerns rather than responding to their needs.

The literature indicates that nurses are used to brushing over sexuality, and that avoidance or referring patients in need of sexual healthcare to other professionals are common strategies used in intimidating situations [35, 43]. The nurses and midwives in this study justify their reluctance by the complexity of sexual healthcare that requires specialisation in the field.

## Conclusion

The study is conducted with a small sample of female participants who reflected mainly on their care of women, although our aim was to address sexual issues related to men and women. Being women, it is likely to be more intimidating to broach the topic with men. Moreover, women are the principal focal point of care for midwives. Yet, the discussion was rich and generated substantive information about nurses’ and midwives’ experience in sexual healthcare and an authentic understanding of the barriers that affect their role. Definitely, it would have been more enriching if we had the occasion to triangulate with a sample of participants who complain of sexual problems. We hope that in future research, we can focus on this aspect. Yet, our aim was to explore nurses and midwives role in sexual health which is not limited to patients with sexual problems. The lack of theoretical information and quasi inexistent training are the overarching concerns of all participants of the study, leaving them poorly qualified to address such a complex and sensitive topic that is socially tabooed and not part of the cultural health practice. These barriers would have been conquered if sexuality was given its due attention in the nursing and midwifery curricula.

## Relevance to clinical practice

Findings emphasise various strategies to improve sexual health, which is at the core of nurses' and midwives' roles and domain of practice. Their commitment to the promotion of peoples’ sexual wellbeing is legitimate. Thus, it is recommended to improve their competencies through a curriculum that provides a solid theoretical and clinical foundation about sexuality and sexual health. This will allow them to be more aware about peoples’ sexual needs and the impact of many health and life conditions on sexual functioning. Being well equipped, they are better placed to overcome their timidity and the taboo around sexuality and feel comfortable in providing sexual healthcare that is evidence-based and culturally sensitive. In this respect, O'Connor and colleagues suggest that an ongoing sexual health training should become an integral part of the continuous professional development [41].

The healthcare system is expected to prioritise sexual health as part of the holistic healthcare approach, provide the necessary resources, and establish standards of sexual healthcare to facilitate sexual health assessment and counselling within an interdisciplinary teamwork. The initiation of awareness and empowerment programmes about sexuality and sexual health would break the silence around the topic and invite people to raise their sexual concerns and seek help. In addition, more research is needed to identify other factors that may affect sexual healthcare provision, considering a more various sample that includes male participants and discuss sexual health issues related to male and female patients of different sexual orientation and marital statuses. This will enhance transferability of findings.

## Data Availability

The datasets used and analyzed during the current study are available from the corresponding author on reasonable request.
